# Supplementary material for: High quality genomes produced from single MinION flow cells clarify polyploid and demographic histories of critically endangered Fraxinus (ash) species
Source: Commun Biol. 2024 Jan 6;7:54. doi: 10.1038/s42003-023-05748-4 (PMC10771460; doi:10.1038/s42003-023-05748-4)
Supplement: Supplementary file 2 — Supplementary Information [file 42003_2023_5748_MOESM2_ESM.pdf]

Title
High quality genomes produced from single MinION flow cells clarify polyploid and demographic histories of critically endangered *Fraxinus* (ash) species

Authors
Steven J. Fleck[1,*], Crystal Tomlin[1], Flavio Augusto da Silva Coelho[1], Michaela Richter[1], Erik S. Danielson[2], Nathan Backenstose[1], Trevor Krabbenhoft[1], Charlotte Lindqvist[1], Victor A. Albert[1,*]

1. Department of Biological Sciences, University at Buffalo, Buffalo, New York 14260, USA
2. Western New York Land Conservancy, East Aurora, NY 14052, USA
* Co-corresponding authors: sjfleck@buffalo.edu, vaalbert@buffalo.edu

Supplementary figures

Figure S1: Raw read length and quality distributions for *Fraxinus* spp..

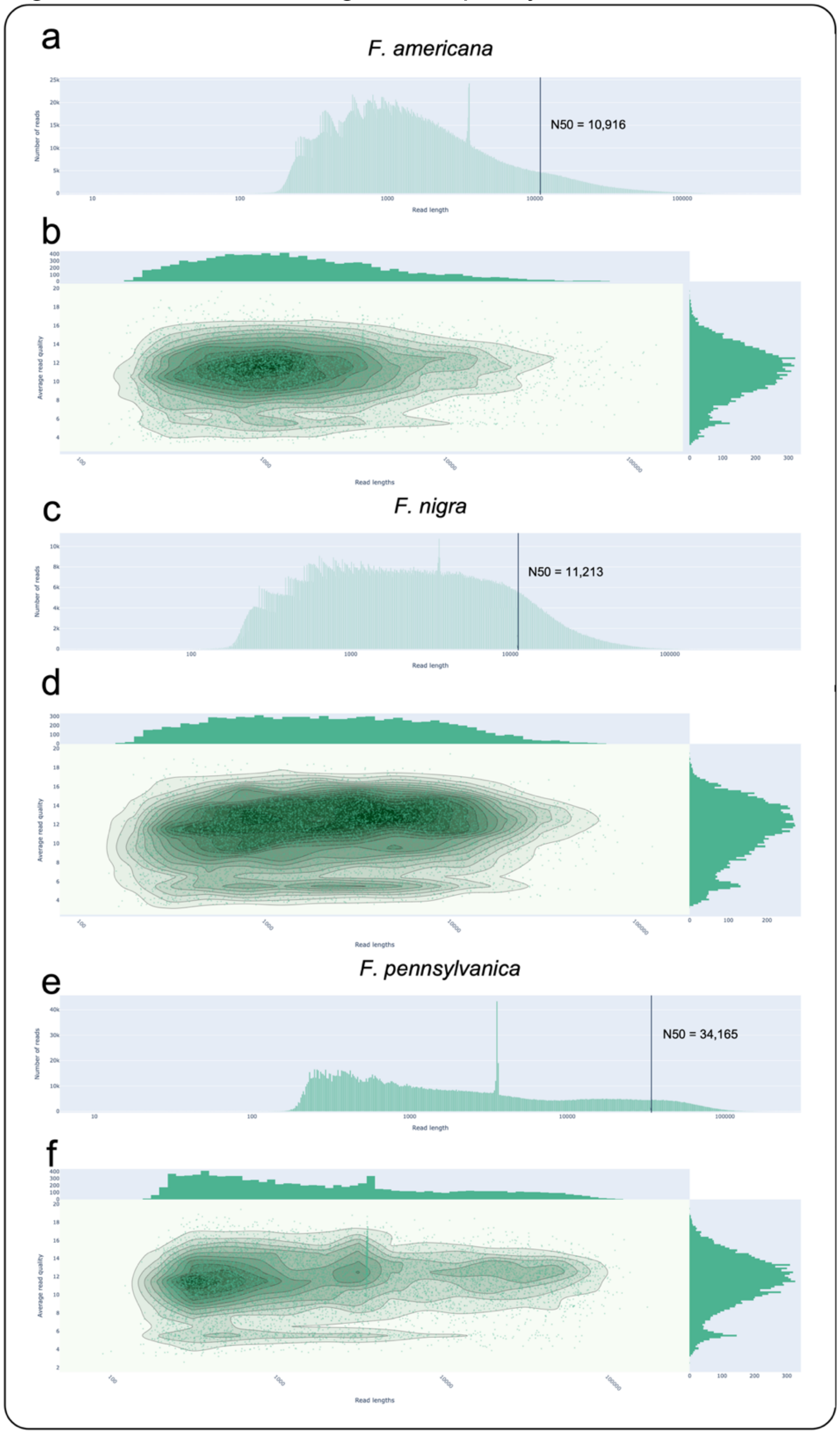

Histogram of read length after log transformation for *Fraxinus americana* (a), *F. nigra* (c), and *F. pennsylvanica* (e). Read N50 is marked on each histogram. Read length vs. average read quality using a kernel density estimation after log transformation of read lengths for *F. americana* (b), *F. nigra* (d), and *F. pennsylvanica* (f). Read length plot above and average quality score histogram to the right of the kernel density estimation plot.

Figure S2: Haploidy assessment of *Fraxinus* genome assemblies before and after running Purge Haplotigs.

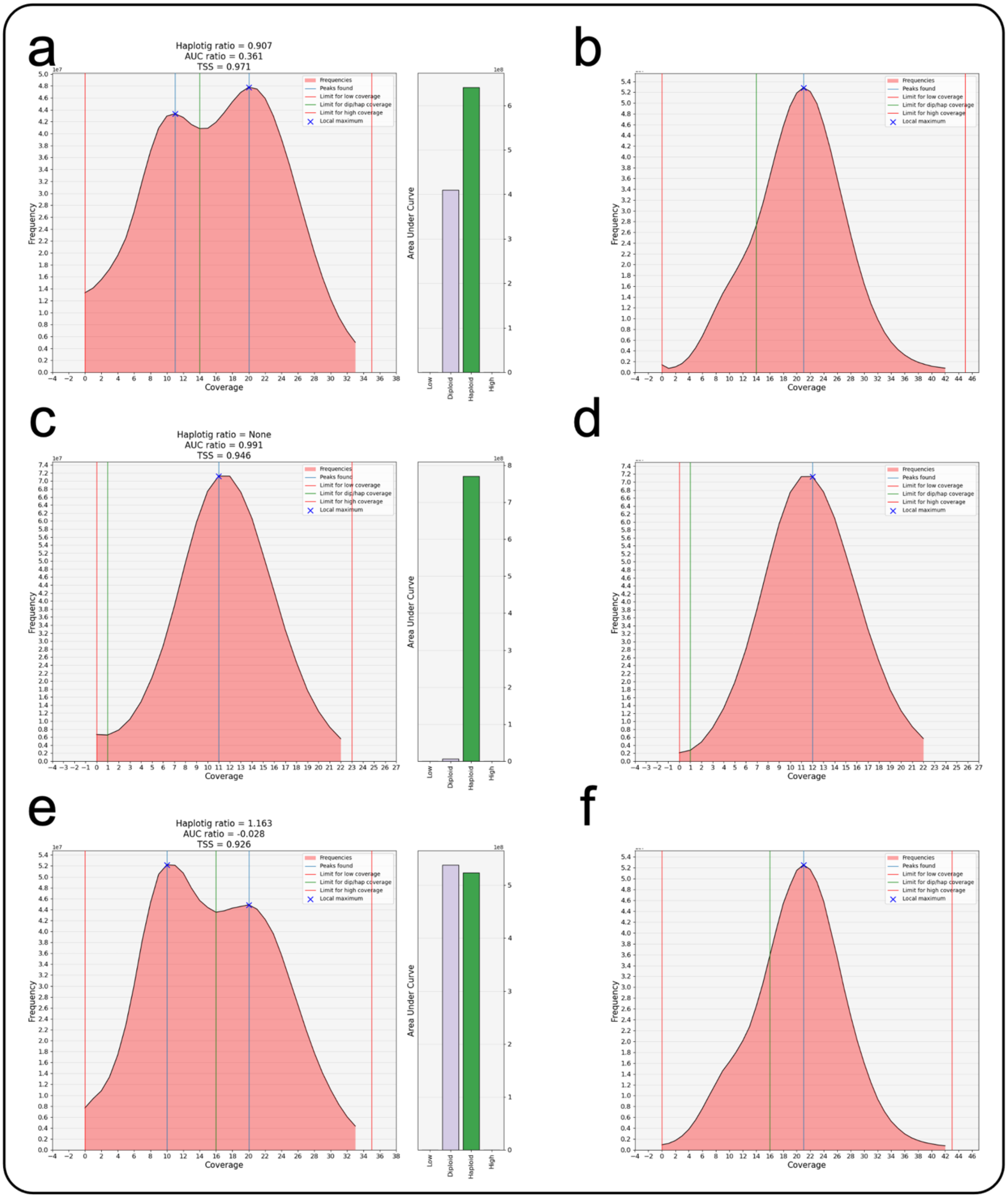

F. americana (a) before and (b) after Purge Haplotigs. *F. nigra* (c) before and (d) after Purge Haplotigs (d). *F. pennsylvanica* (e) before and (f) after Purge Haplotigs. AUC ratio = 1 - (D/H); TSS = 1 - (abs(G - (H + D/2)) / G). Abbreviations: AUC = area under curve, D = diploid area under curve, H = haploid area under curve, TSS = total size score, G = estimated genome size.

Figure S3: Syntenic regions and frequency plots for synonymous substitution rates (Ks) between syntenic CDS pairs for each partially diploid *Fraxinus* assembly and itself.

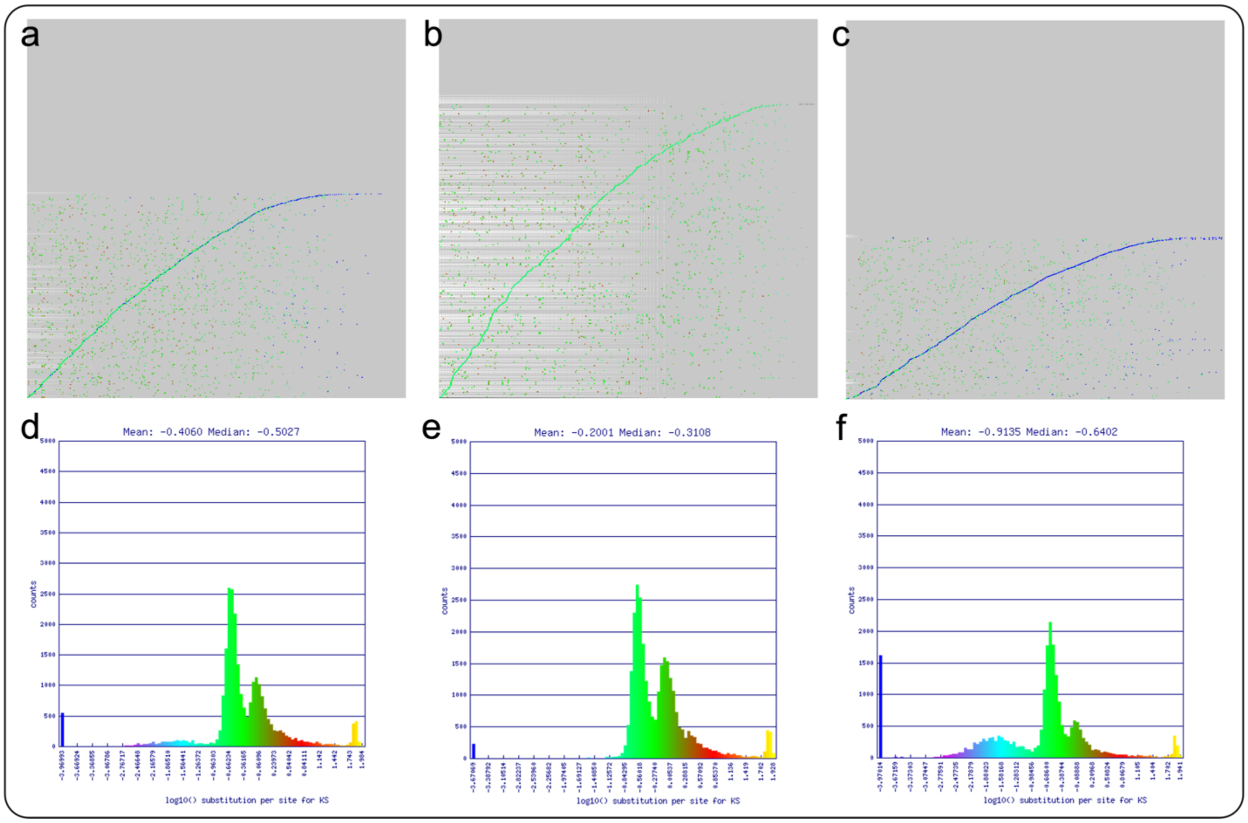

(a&d) Self-vs-self syntenic dot plots for *F. americana*, (b&e) *F. nigra*, and (c&f) *F. pennsylvanica*. Colors in Ks histograms correspond with colors of each point in the dot plots.

Figure S4: Syntenic regions and frequency plots for synonymous substitution rates (Ks) between syntenic CDS pairs for each haploid *Fraxinus* assembly and itself.

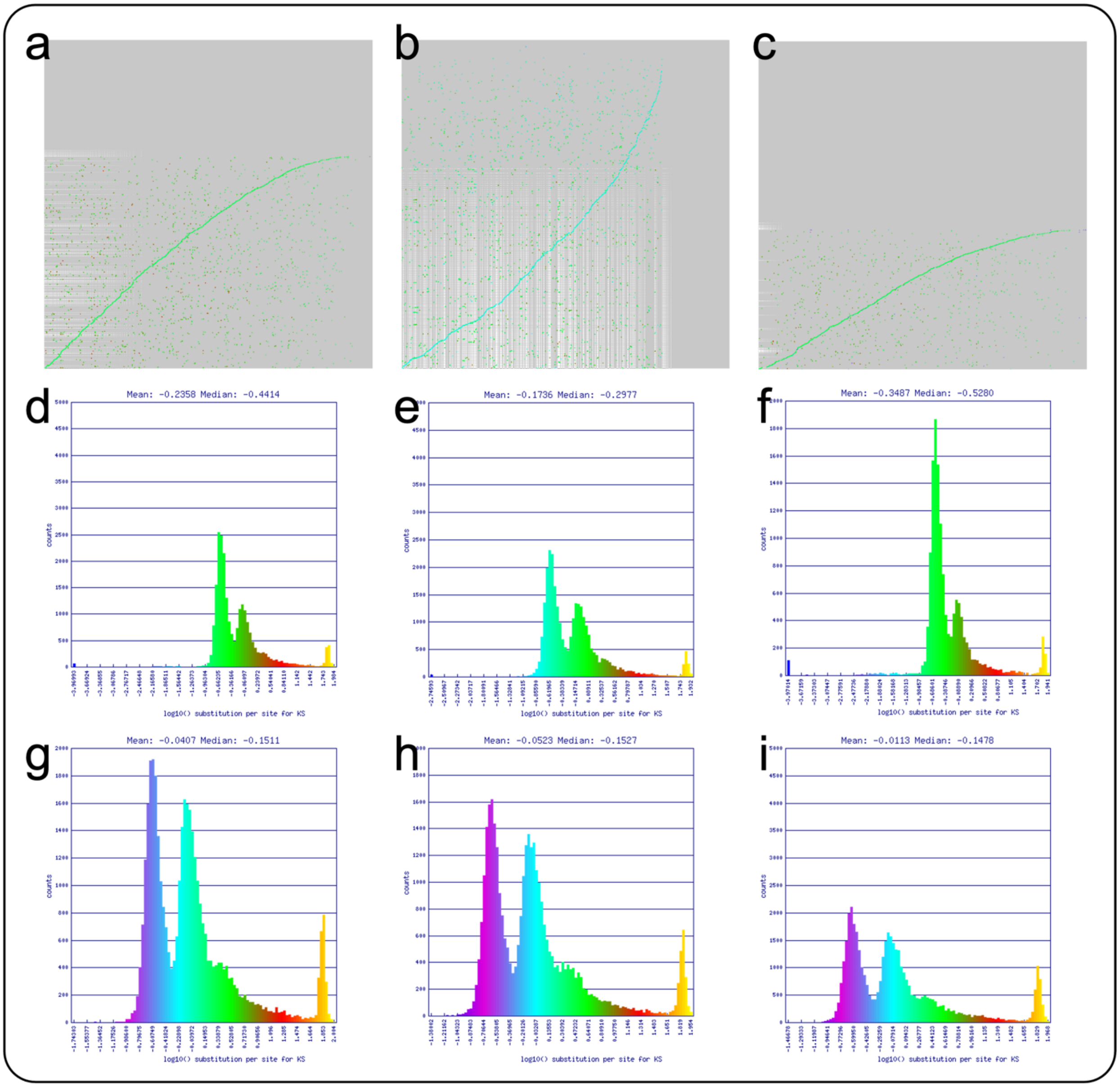

Self-self syntenic dot plots for (a&d) *F. americana*, (b&e) *F. nigra*, and (c&f) *F. pennsylvanica*. Colors in Ks histograms correspond with colors of each point in the dot plots. Ks frequency plots for Huff et al.[1] assemblies are also included for comparison: (g) *F. americana*-v0.2.1, (h) *F. nigra*-v0.2.1, and (i) *F. pennsylvanica*-v1.4.

Figure S5: Unpurged heterozygosity in previously published *Osmanthus fragrans* and *Olea europaea* assemblies.

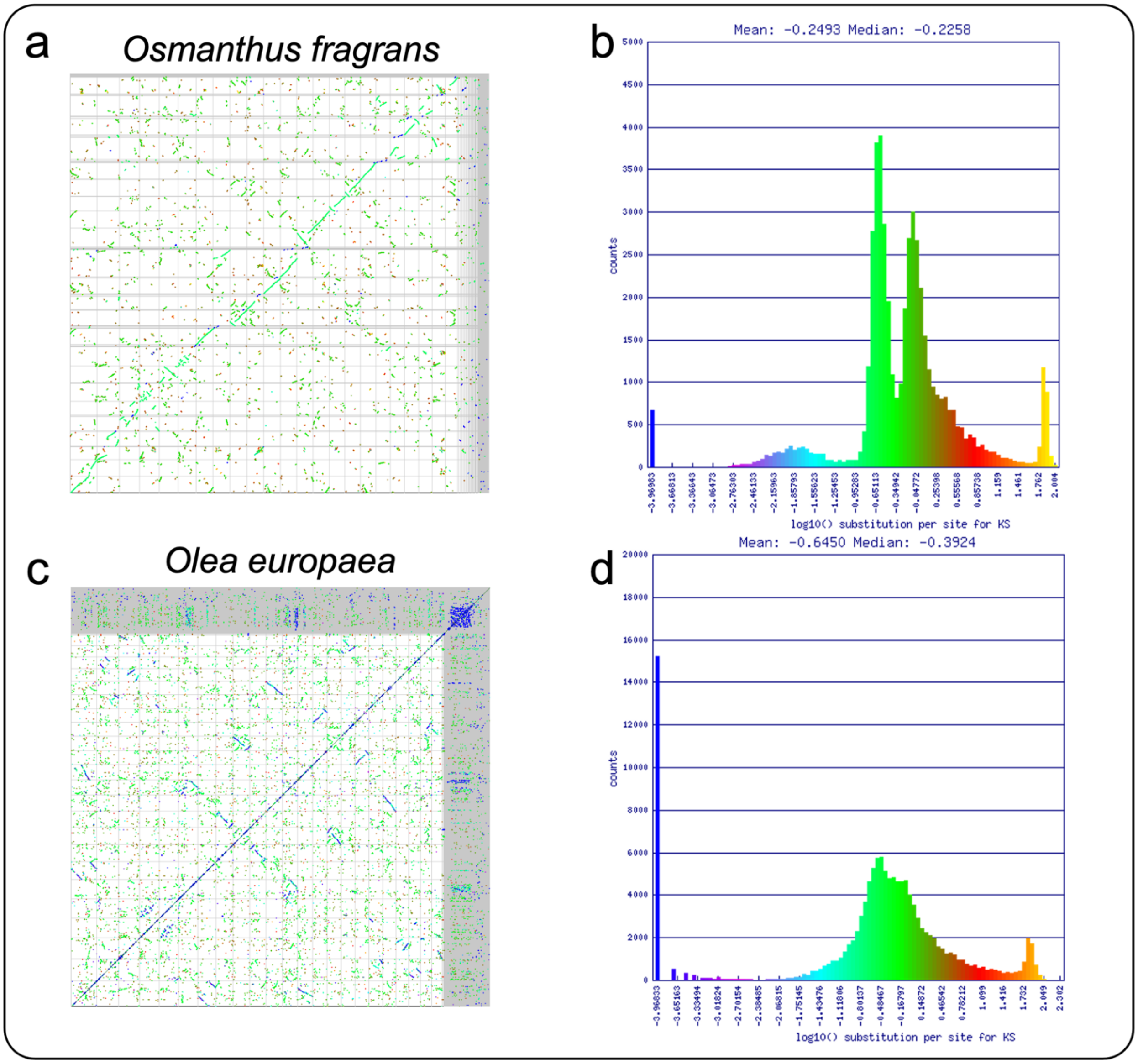

Self-vs-self syntenic dot plot for (a) *O. fragrans* and (c) *O. europaea*. Full genome runs along x and y-axis. Each dot represents a syntenic gene pair between paralogs within each genome. Dot color correlates with synonymous substitution rate (Ks) values (in $\log_{10}$) in accompanying histograms: (b) *O. fragrans* and (d) *O. europaea*.

Figure S6: One-to-one macrosynteny relationships among members of the Oleeae tribe.

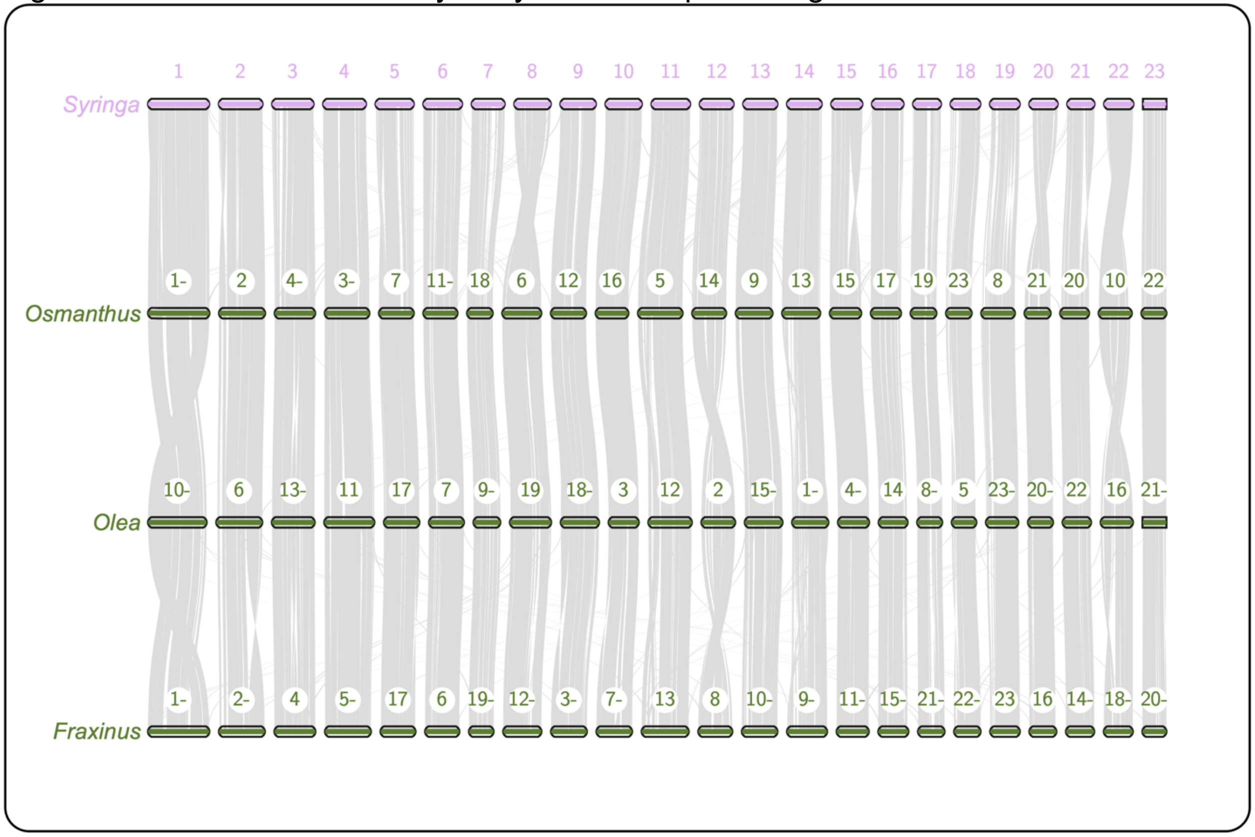

Macrosynteny on a chromosome-scale between *Syringa oblata*, *Osmanthus fragrans*, *Olea europaea*, and the *Fraxinus nigra* RagTag assembly. Numbers above chromosomes indicate chromosome number and reversed sequences are followed by "-". Syntenic blocks between *Syringa*, *Osmanthus*, *Olea*, and *Fraxinus* chromosomes are connected by light grey lines.

Figure S7. Syntenic dot plot, fractionation bias, and macrosynteny between jasmine and grapevine.

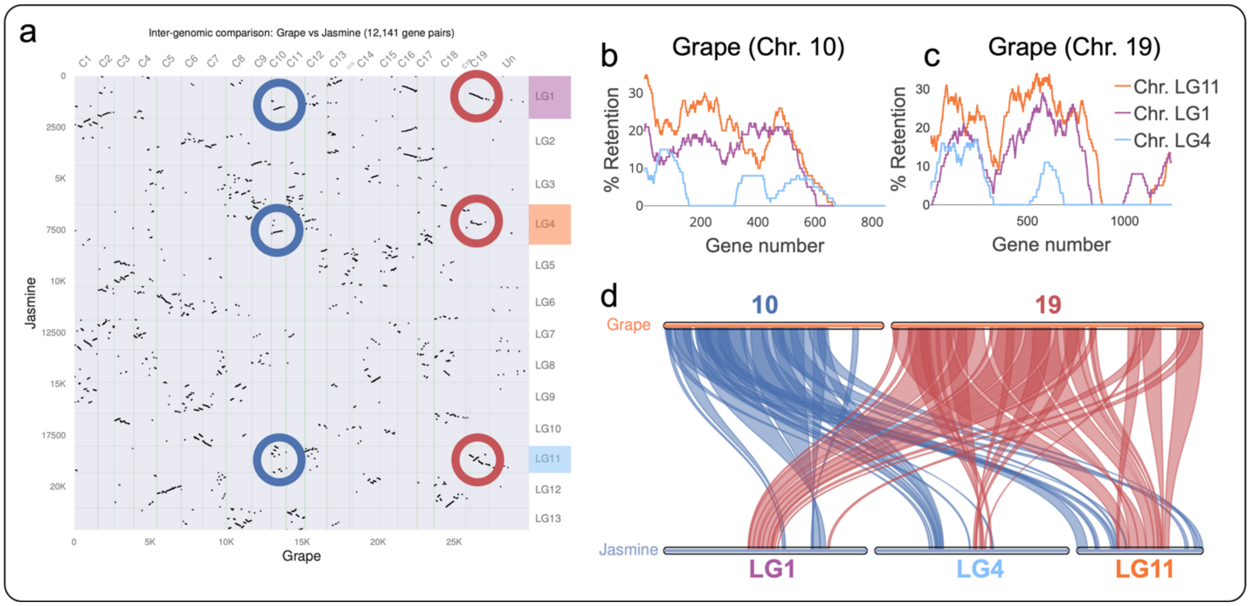

(a) Syntenic dot plot with grapevine on the x-axis and jasmine on the y-axis. Each dot represents a syntenic gene pair between the two assemblies. Blue circles highlight the syntenic dots between grapevine chromosomes 10 and jasmine chromosomes 1, 4, and 11. Red circles highlight the syntenic dots between grapevine chromosomes 19 and jasmine chromosomes 1, 4, and 11. (b&c) Gene retention of syntenic gene blocks between grapevine and jasmine. Y-axis represents the percent of gene retention of each jasmine syntenic block compared with windows of genes on grapevine chromosomes 10 and 19. Line color correlates with query chromosome number in the key and the highlighted chromosome names in (a). (d) Macrosyntenic blocks between grapevine chromosomes 10 and 19 and jasmine chromosome 1, 4, and 11. The colors of the connecting lines correlate to the circled syntenic dots in (a).

Figure S8. Syntenic dot plot, syntenic depth, and macrosynteny between jasmine and forsythia.

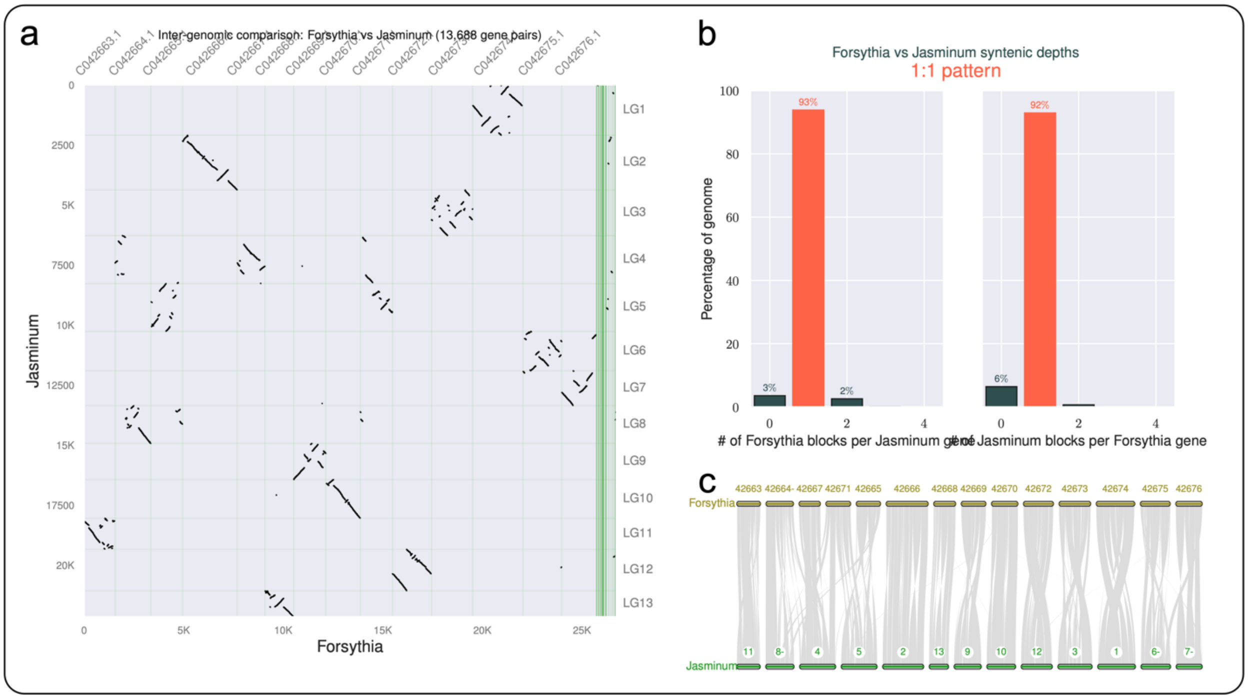

(a) Syntenic dot plot with forsythia on the x-axis and jasmine on the y-axis. Each black dot represents a syntenic gene pair between the two assemblies. (b) 1:1 syntenic depth between forsythia and jasmine. (c) The fourteen chromosomes of forsythia show a 1:1 ratio with jasmine's thirteen chromosomes. Grey connectors join syntenic blocks between the two assemblies. Numbers above chromosomes indicate chromosome name.

Figure S9. Syntenic dot plot and fractionation bias between *F. americana* and *J. sambac*.

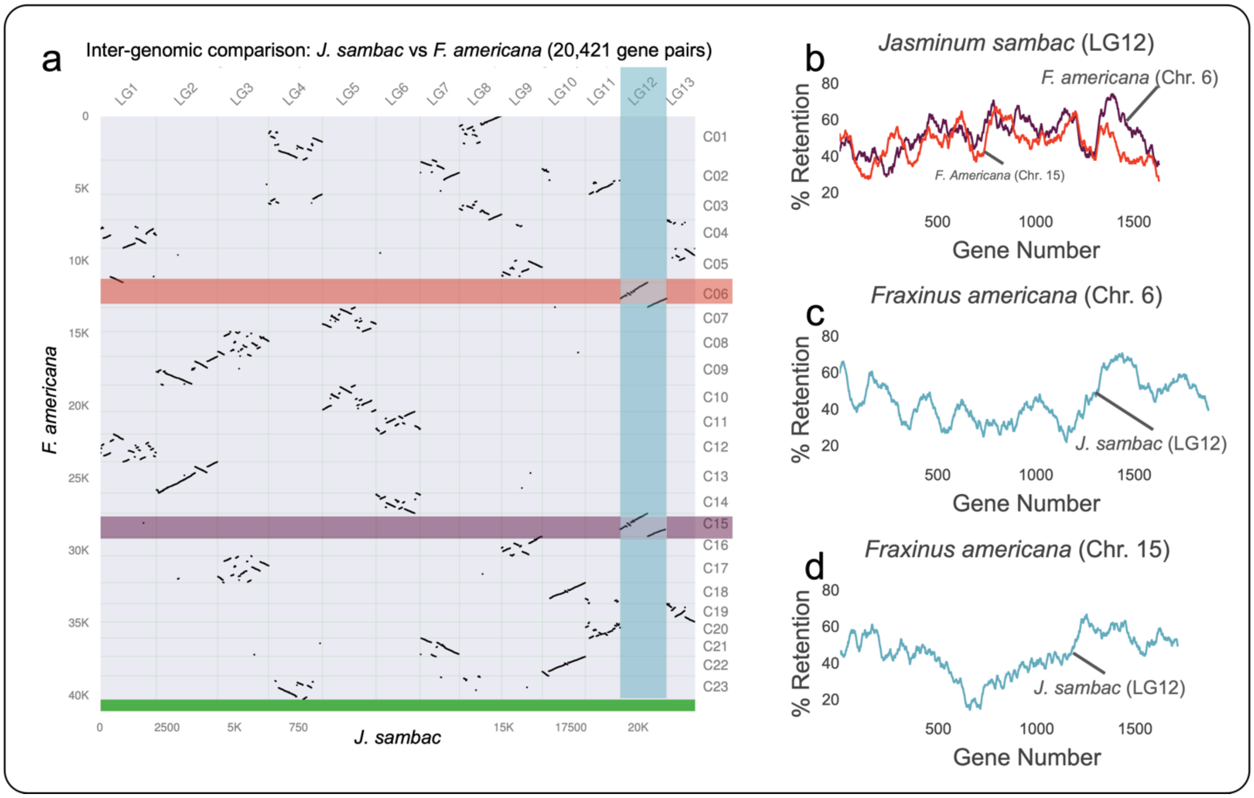

(a) Syntenic dot plot with jasmine on the x-axis and *F. americana* RagTag assembly on the y-axis. Each block dot represents a syntenic gene pair between the two assemblies. Colored rectangles highlight the syntenic dots between Jasmine chromosome 12 and *F. americana* chromosomes 6 and 15. Colors of rectangles correlate with line color in (b), (c), and (d). (b) Fractionation bias plot showing two *F. americana* chromosomes (chr. 6 and 15) with similar patterns of gene retention when compared to one *J. sambac* chromosome (LG12). (c&d) One *J. sambac* chromosome (LG12) showing high gene retention with two *F. americana* chromosomes (chr. 6 and 15). Lines representing *F. americana* chromosome 6 and 15 and *J. sambac* chromosome 12 are labeled.

Figure S10: Pairwise Sequentially Markovian Coalescent with R (PSMCR) paleodemographic analyses for *Fraxinus* species.

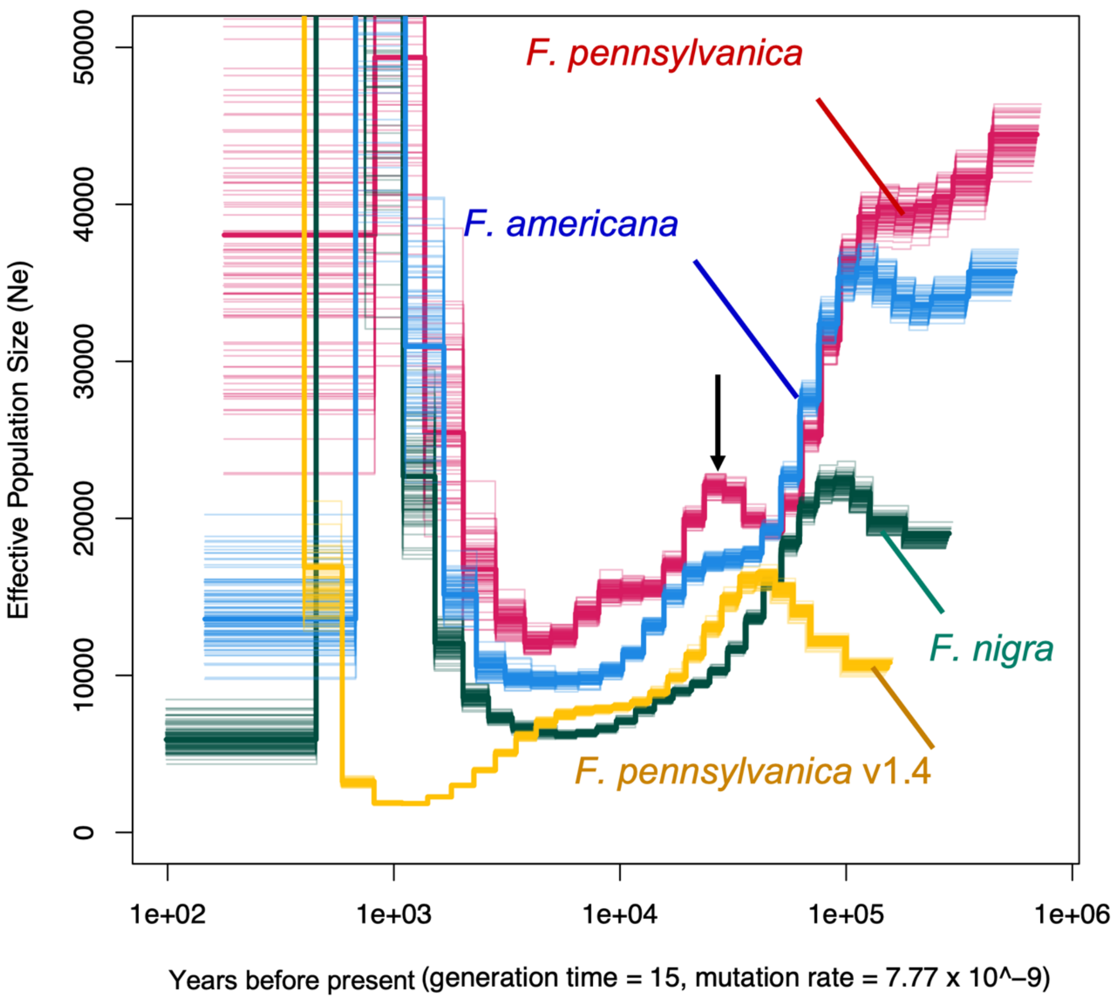

Demographic curve colors represent the following genome assemblies: blue: *F. americana*; green: *F. nigra*; pink: *F. pennsylvanica*; yellow: *F. pennsylvanica* reference assembly (v1.4). PSMCR plots were generated by mapping *F. americana*, *F. nigra*, and *F. pennsylvanica* against their own ONT long-reads and the *F. pennsylvanica* reference assembly mapped against its Illumina short-reads and filtered to the same coverage as the others. All curves assume a mutation rate of 7.77e-9 and a generation time of 15 years. The bcftools multiallelic and rare-variant calling model was used and maxt was set to 10 for PSMCR. Thin black arrow points to the hump in our *F. pennsylvanica*'s demographic curve.

Figure S11: Pairwise sequentially markovian coalescent with R (PSMCR) representing the long-read and short-read *Fraxinus pennsylvanica* assemblies.

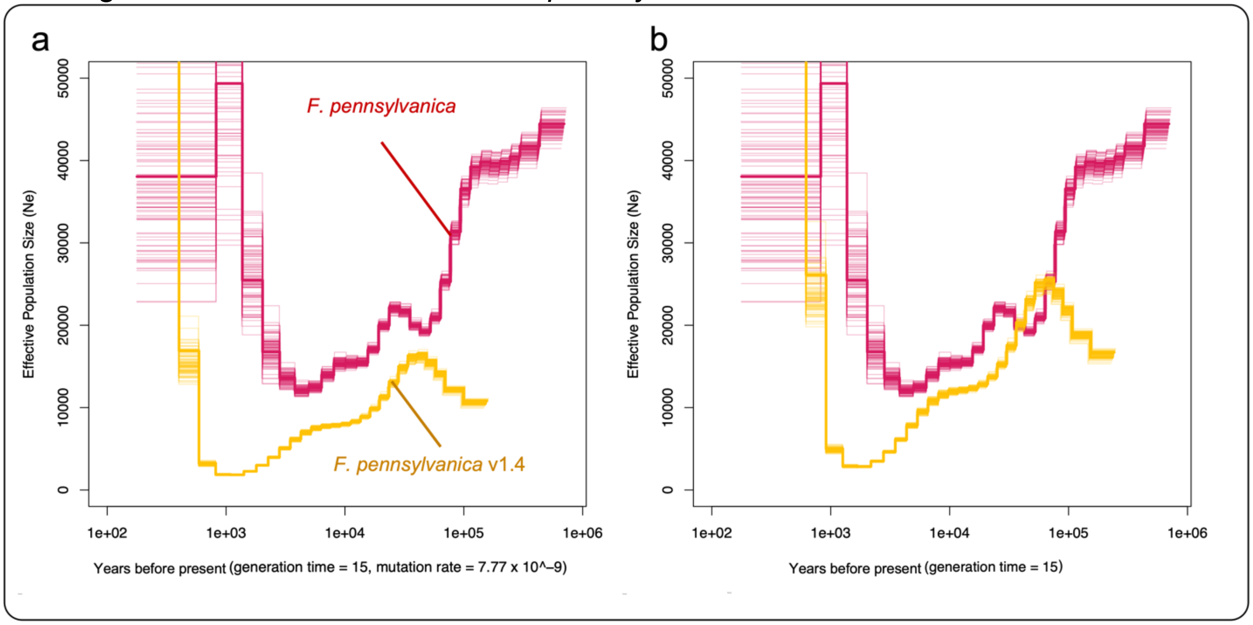

(a) PSMCRs created using our *F. pennsylvanica* assembly and ONT long reads (pink) and Illumina short reads from the *F. pennsylvanica* reference assembly (yellow). Each curve assumes a mutation rate of 7.77e-9 and a generation time of 15 years. (b) Same as (a), but the short-read PSMC curve has its mutation rate lowered to 5.03e-9 using the same formula used to adjust the long-read PSMCs in Figure 2a (Table S9). Effective population size (Ne) is on the y-axis and time in years is on the x-axis. The bcftools multiallelic and rare-variant calling model was used and maxt was set to 10 for PSMCR.

Figure S12: Pairwise sequentially markovian coalescent with R (PSMCR) for *Fraxinus pennsylvanica*.

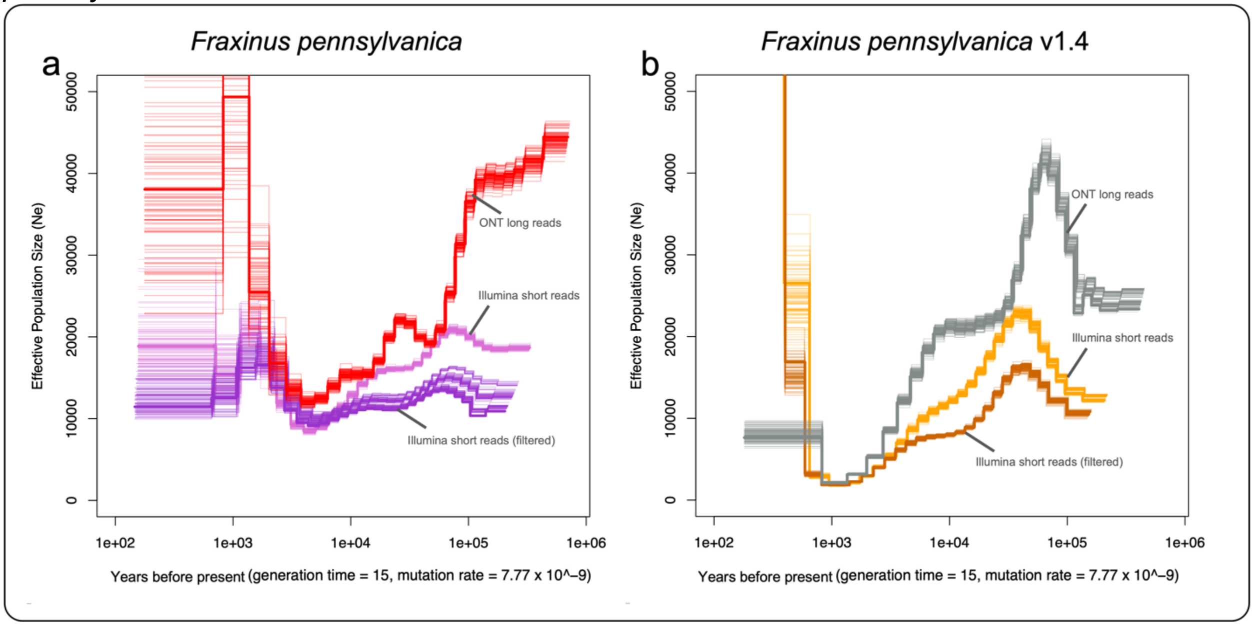

(a) PSMCRs created using our *F. pennsylvanica* assembly and ONT long reads (red), Illumina short reads from the *F. pennsylvanica* reference assembly (light purple), and Illumina short reads from the *F. pennsylvanica* reference assembly that has been filtered to match the depth of coverage of the long-read mapping (dark purple). (b) PSMCs created using the *F. pennsylvanica* reference assembly and its own Illumina short reads (orange), its own Illumina short reads that have been filtered to match the depth of coverage of the long-read mapping (dark orange), and the ONT long reads from our *F. pennsylvanica* sample (grey). Effective population size (Ne) is on the y-axis and time in years is on the x-axis. The bcftools multiallelic and rare-variant calling model was used and maxt was set to 10 for PSMCR.

Figure S13: Species trees based on OrthoFinder data.

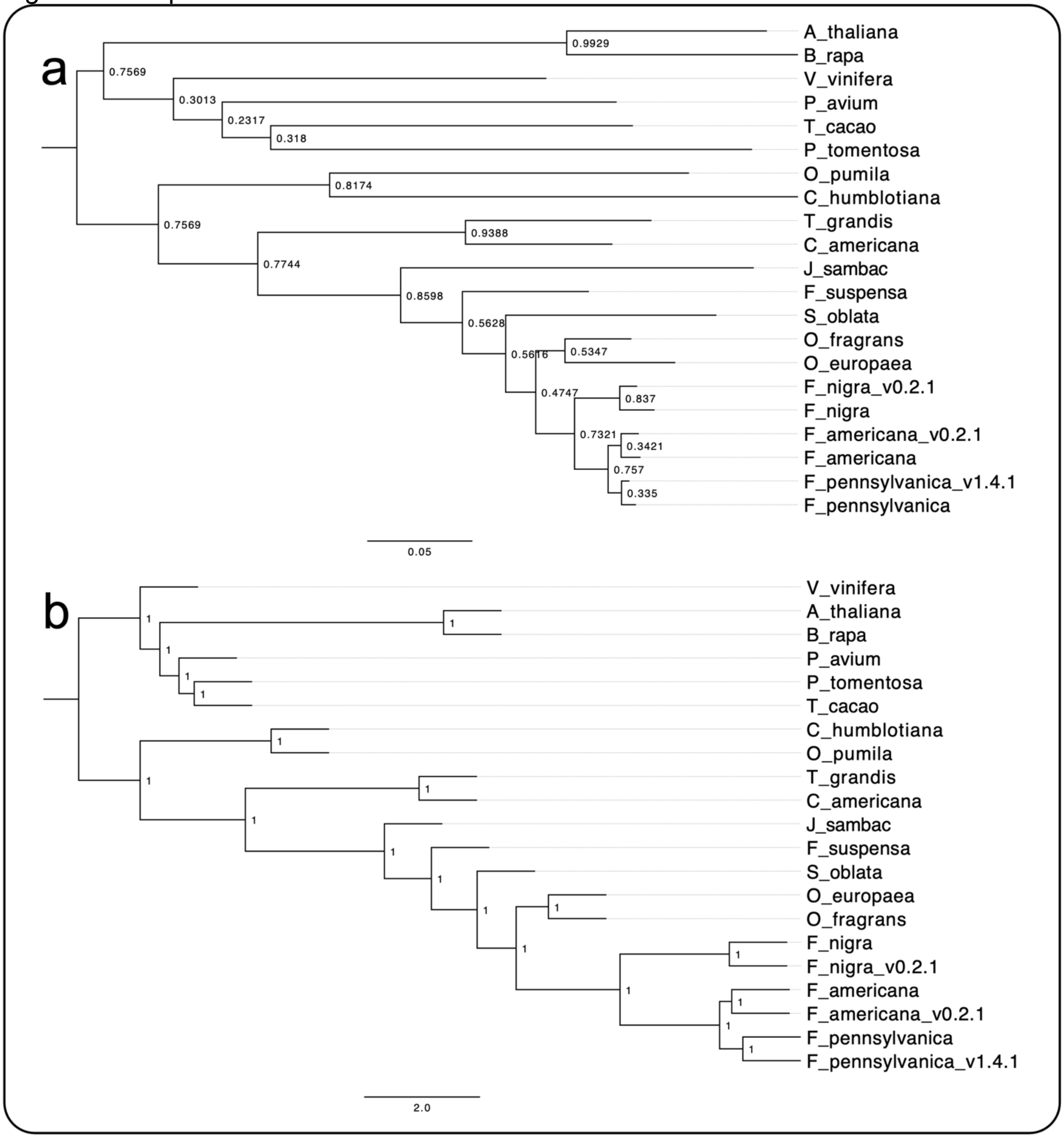

(a) OrthoFinder species tree. Branch support is derived from the proportion of species trees derived from single-locus gene trees supporting each bipartition using STAG. Branch lengths of the STAG tree represent average substitutions per site among orthogroup input trees. (b) ASTRAL tree generated using single-copy gene trees from OrthoFinder. Internal branch lengths are in coalescent units and branch support is measured as local posterior probabilities. Tip labels are as follows: (Lamiales, Oleaceae) F_pennsylvanica: *Fraxinus pennsylvanica*; F_pennsylvanica_v1.4.1: *F. pennsylvanica* reference assembly (Huff et al.[1]); F_americana: *Fraxinus americana*; F_americana_v0.2.1: *Fraxinus americana* (Huff et al.[1]); F_nigra: *Fraxinus nigra*;

F_nigra_v0.2.1: *Fraxinus nigra* (Huff et al.[1]); O_fragrans: *Osmanthus fragrans*; O_europaea: *Olea europaea*; S_oblata: *Syringia oblata*; F_suspensa: *Forsythia suspensa*; J_sambac: *Jasminum sambac*; (other Lamiales) C_amerciana: *Callicarpa americana*; T_grandis: *Tectona grandis*; (Gentianales) C_humblotiana: *Coffea humblotiana*; O_pumila: *Ophiorrhiza pumila*; (Rosids) T_cacao: *Theobroma cacao*; P_tomentosa: *Populus tomentosa*; P_avium: *Prunus avium*; B_rapa: *Brassica rapa*; A_thaliana: *Arabidopsis thaliana*; V_vinifera: *Vitis vinifera*.

Figure S14: Syntenic regions and frequency plots for synonymous substitution rates (Ks) between syntenic CDS pairs for each haploid *Fraxinus* assembly and *Vitis vinifera*.

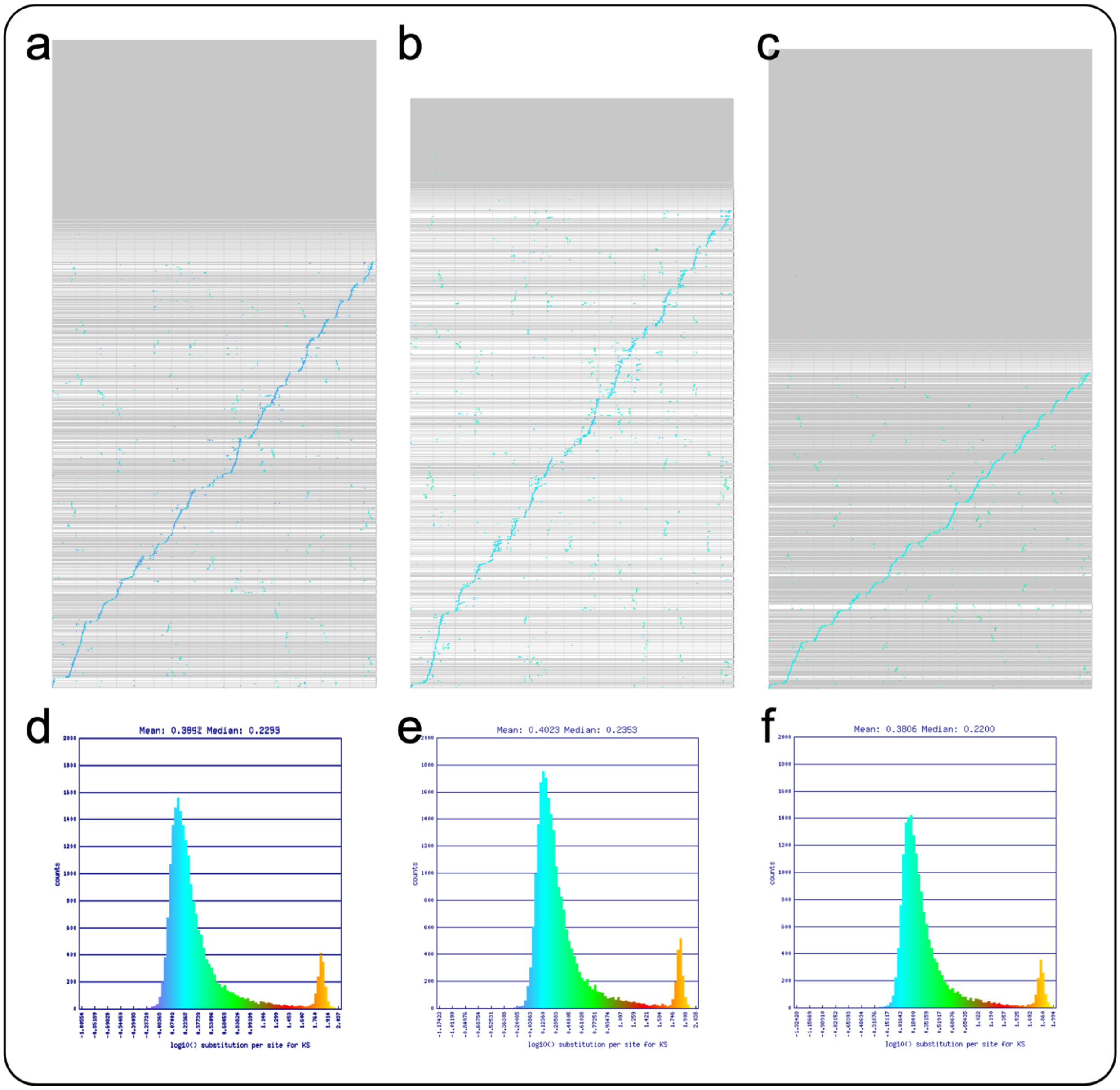

Syntenic dot plots for *V. vinifera* against (a) *F. americana*, (b) *F. nigra*, and (c) *F. pennsylvanica*. Dot color correlates with synonymous substitution rate (Ks) values (in $\log_{10}$) in accompanying histograms: (d) *F. americana*, (e) *F. nigra*, and (f) *F. pennsylvanica*.

Figure S15: Syntenic regions and frequency plots for synonymous substitution rates (Ks) between syntenic CDS pairs for each haploid *Fraxinus* assembly and the *F. pennsylvanica* reference assembly.

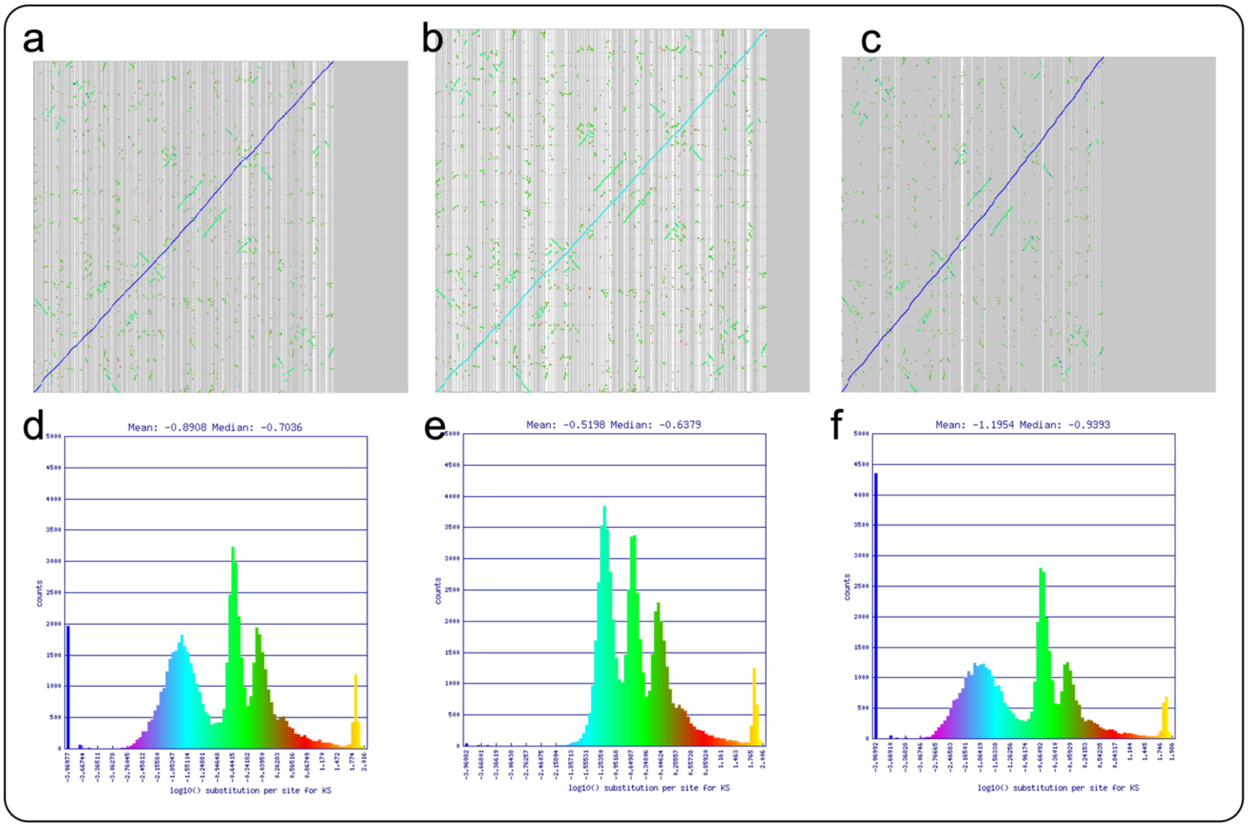

Syntenic dot plots for the *F. pennsylvanica* reference assembly against (a) *F. americana*, (b) *F. nigra*, and (c) *F. pennsylvanica*. Dot color correlates with synonymous substitution rate (Ks) values (in $\log_{10}$) in accompanying histograms: (d) *F. americana*, (e) *F. nigra*, and (f) *F. pennsylvanica*.

Figure S16: Read depth histograms produced by Purge Haplotigs.

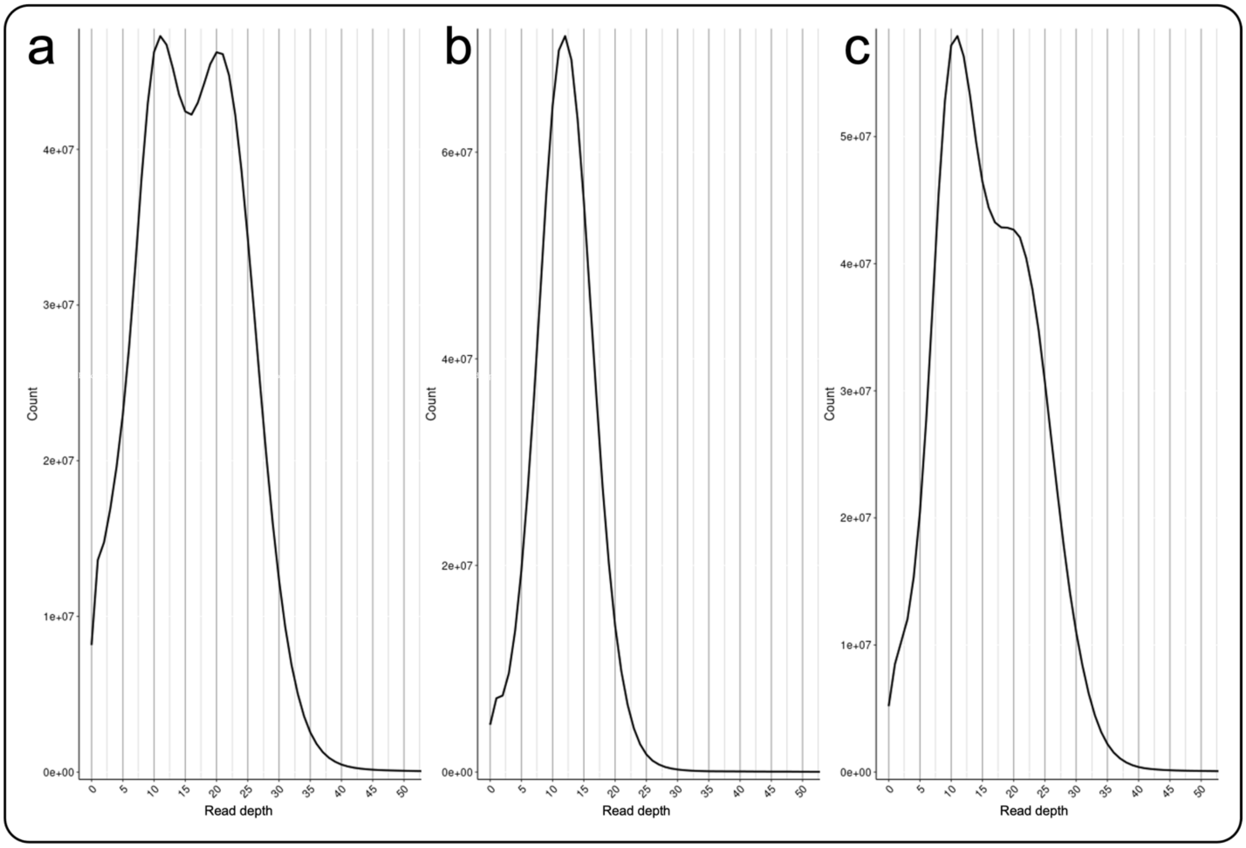

Depth of reads mapped against corresponding primary Flye assemblies for (a) *F. americana*, (b) *F. nigra*, and (c) *F. pensylvanica*. Read depth is on the x-axis and frequency is on the y-axis.

Table S1: Nanoplot sequencing stats for *Fraxinus* spp.

Sample	*Fraxinus americana*	*Fraxinus nigra*	*Fraxinus pennsylvanica*
Mean read length	3,600.80	4,893.90	7,735.80
Mean read quality	10.9	11.2	11.4
Median read length	1,266	2,106	1,319
Median read quality	11.1	11.6	11.7
Number of reads	6,065,809	3,788,248	2,581,752
Read length N50	10,916	11,213	34,165
STDEV read length	7,857.10	7,447.30	15,418.20
Total bases	21,841,746,309	18,539,126,624	19,971,958,290
Number, percentage and megabases of reads above quality cutoffs			
>Q5	5,930,147 (97.8%) 21,585.6Mb	3,674,454 (97.0%) 18,219.9Mb	2,535,016 (98.2%) 19,830.2Mb
>Q7	5,497,205 (90.6%) 20,368.9Mb	3,325,444 (87.8%) 16,891.4Mb	2,357,593 (91.3%) 18,979.0Mb
>Q10	3,975,554 (65.5%) 15,529.1Mb	2,564,692 (67.7%) 13,685.5Mb	1,865,938 (72.3%) 15,784.9Mb
>Q12	2,194,311 (36.2%) 8,495.9Mb	1,706,471 (45.0%) 9,190.7Mb	1,170,738 (45.3%) 10,245.7Mb
>Q15	310,054 (5.1%) 755.8Mb	348,295 (9.2%) 1,526.5Mb	208,967 (8.1%) 796.1Mb
Top 5 highest mean basecall quality scores and their read lengths			
1	90.0 (258)	39.2 (398)	31.4 (316)
2	90.0 (250)	28.7 (466)	30.4 (888)
3	25.5 (236)	26.2 (287)	28.0 (240)
4	24.9 (3,516)	25.8 (269)	26.8 (249)
5	23.3 (366)	24.4 (162)	26.8 (268)
Top 5 longest reads and their mean basecall quality score			
1	634,799 (6.3)	659,014 (3.6)	300,359 (5.5)
2	316,710 (4.7)	654,569 (3.6)	275,810 (8.8)
3	288,023 (11.4)	539,167 (3.6)	271,844 (9.7)
4	268,430 (10.7)	434,091 (4.1)	256,584 (11.8)
5	268,167 (12.9)	356,372 (3.6)	253,696 (9.6)

Table S2: Assembly and annotation stats for *Fraxinus* spp. primary Flye assemblies. *F. pennsylvanica* v1.4 is the chromosome-level assembly by Huff et al.[1]

Assembly	*F. americana*	*F. nigra*	*F. pennsylvanica*	*F. pennsylvanica* v1.4
# contigs	13,096	3,964	13,855	110
Largest contig	3,688,385	6,590,250	6,486,905	56,547,140
Est. Total length	875 Mbp	829 Mbp	869 Mbp	869 Mbp
Total length	1,075,956,968	795,919,879	1,081,813,881	756,791,283
GC (%)	35.31	34.78	35.21	34.40%
N50	369,422	1,037,385	196,920	33,221,578
L50	708	215	1,436	10
# N's per 100 kbp	0.67	0.46	0.57	12,120.86
Complete BUSCOs	1565 (97.0%)	1561 (96.7%)	1565 (97.0%)	1576 (97.6%)
Complete single-copy BUSCOs	1146 (71.0%)	1293 (80.1%)	1065 (66.0%)	1308 (81.0%)
Complete duplicated BUSCOs	419 (26.0%)	268 (16.6%)	500 (31.0%)	268 (16.6%)
Fragmented BUSCOs	35 (2.2%)	25 (1.5%)	33 (2.0%)	26 (1.6%)
Missing BUSCOs	14 (0.8%)	28 (1.8%)	16 (1.0%)	12 (0.8%)
Total BUSCOs searched	1614	1614	1614	1614
Annotation	*F. americana*	*F. nigra*	*F. pennsylvanica*	*F. pennsylvanica* v1.4
gene model/mRNA count	49,500/55,584	38,374/43,707	50,130/56,118	35,470/35,470
Complete BUSCOs	97.4% (1572)	97.4% (1572)	98.1% (1583)	82.5% (1332)
Complete single-copy BUSCOs	70.0% (1130)	79.9% (1290)	66.0% (1065)	70.8% (1142)
Complete duplicated BUSCOs	27.4% (442)	17.5% (282)	32.1% (518)	11.8% (190)
Fragmented BUSCOs	0.9% (15)	0.8% (13)	0.9% (14)	2.4% (38)
Missing BUSCOs	1.7% (27)	1.8% (29)	1.1% (17)	15.1% (244)
Total BUSCOs searched	1614	1614	1614	1614

Table S3: Repetitive element statistics for *Fraxinus* spp. primary Flye assemblies

Assembly	*Fraxinus americana*			*Fraxinus nigra*			*Fraxinus pennsylvanica*		
sequences:	13,098			3,966			13,858		
total length:	1,075,957,570 bp (1,075,950,370 excl N/X-runs)			795,920,351 bp (795,916,651 excl N/X-runs)			1,081,815,219 bp (1,081,809,019 bp excl N/X-runs)		
GC level:	35.31%			34.78%			35.21%		
bases masked:	634,195,562 bp (58.94%)			467,015,253 bp (58.68%)			637,543,510 bp (58.93%)		
	# of elements*	length occupied (bp)	% of sequence	# of elements*	length occupied (bp)	% of sequence	# of elements*	length occupied (bp)	% of sequence
Retroelements	384,492	334,371,432	31.08%	270,365	249,099,175	31.30%	399,821	337,941,412	31.24%
SINEs:	2,550	372,349	0.03%	3,439	551,054	0.07%	7,160	1,170,284	0.11%
Penelope	-	-	0.00%	-	-	0%	-	-	0%
LINEs:	21,726	13,239,449	1.23%	14,017	8,510,589	1.07%	22,429	13,614,187	1.26%
CRE/SLACS	-	-	0.00%	-	-	0%	-	-	0%
L2/CR1/Rex	490	553,009	0.05%	177	37,105	0%	599	121,452	0.01%
R1/LOA/Jockey	195	22,167	0.00%	-	-	0%	126	13,072	0%
R2/R4/NeSL	146	40,161	0.00%	612	153,912	0.02%	-	-	0%
RTE/Bov-B	7,807	1,376,305	0.13%	5,235	1,122,283	0.14%	8,667	1,484,648	0.14%
L1/CIN4	11,680	10,865,949	1.01%	7,536	7,147,641	0.90%	10,942	11,256,441	1.04%
LTR elements:	360,216	320,759,634	29.81%	252,909	240,037,532	30.16%	370,232	323,156,941	29.87%
BEL/Pao	2,508	1,284,160	0.12%	-	-	0%	633	308,335	0.03%
Ty1/Copia	176,262	148,750,776	13.82%	128,129	113,851,137	14.30%	195,982	154,958,003	14.32%
Gypsy/DIRS1	157,339	145,420,711	13.52%	113,663	111,284,487	13.98%	155,368	144,758,966	13.38%
Retroviral	-	-	0.00%	440	159,467	0.02%	96	12,804	0%
DNA transposons	169,402	60,540,713	5.63%	118,207	43,467,790	5.46%	172,354	58,779,532	5.43%
hobo-Activator	82,312	29,932,752	2.78%	61,077	22,912,804	2.88%	96,418	32,993,389	3.05%
Tc1-IS630-Pogo	1,828	583,760	0.05%	4,760	1,157,223	0.15%	3,296	1,021,611	0.09%
En-Spm	-	-	0.00%	-	-	0%	-	-	0%
MuDR-IS905	-	-	0.00%	-	-	0%	-	-	0%
PiggyBac	-	-	0.00%	-	-	0%	-	-	0%
Tourist/Harbinger	18,624	5,639,384	0.52%	9,295	2,501,107	0.31%	21,810	5,454,896	0.50%
Other**	-	-	0.00%	-	-	0%	-	-	0%
Rolling-circles	16,660	10,999,733	1.02%	6,535	7,764,158	0.98%	12,684	7,995,087	0.74%
Unclassified:	988,632	217,281,568	20.19%	709,256	158,801,314	19.95%	1,022,970	220,782,260	20.41%
Total interspersed rep	612,193,713		56.90%	451,368,279		56.71%	617,503,204		57.08%
Small RNA:	7,032	1,396,645	0.13%	5,409	1,074,683	0.14%	12,890	2,517,040	0.23%
Satellites:	3,997	763,825	0.07%	492	115,146	0.01%	6,396	857,697	0.08%
Simple repeats:	181,069	7,715,883	0.72%	142,655	5,925,114	0.74%	186,687	8,264,036	0.76%
Low complexity:	31,623	1,498,112	0.14%	23,241	1,088,970	0.14%	32,065	1,576,730	0.15%

*most repeats fragmented by insertions or deletions have been counted as one element

**Mirage, P-element, Transib

Table S4: Ploidy statistics for Fraxinus spp. primary Flye assemblies generated from HapPy (Fig. S2A, C, E).

	Flye Assemblies before PurgeHaplotigs		
	Fraxinus americana	*Fraxinus nigra*	*Fraxinus pennsylvanica*
Haploid (H) area under curve (AUC)	641,144,862	770,860,918	523,829,163
Diploid (D) area under curve (AUC)	409,767,110	6,659,091	538,718,054
Ratio: 1-(D/H)	0.360882175	0.991361488	-0.028423181
Haploidy: H/(H + (D/2))	75.78%	99.57%	66.04%

Table S5: Repetitive element statistics for *Fraxinus* spp. haploid assemblies compared with Fraxinus spp. assemblies generated in Huff et al.[1]

Assembly	*F. americana*			*F. americana* (Huff et al.)			*F. nigra*		
sequences:	4,365			185,071			2,546		
total length:	851,858,583 bp (851,851,783 bp excl N/X-runs)			659,772,997 bp (642,548,809 bp excl N/X-runs)			776,258,641 bp (776,254,941 bp excl N/X-runs)		
GC level:	35.26%			34.43%			34.76%		
bases masked:	497,843,398 bp (58.44%)			313,817,079 bp (47.56%)			454,976,171 bp (58.61%)		
	# of elements*	length occupied (bp)	% of sequence	# of elements*	length occupied (bp)	% of sequence	# of elements*	length occupied (bp)	% of sequence
Retroelements	275,610	267,822,293	31.44%	362,514	124,150,677	18.82%	261,772	240,099,738	30.93%
SINEs:	3,535	547,506	0.06%	2,359	306,359	0.05%	2,178	308,864	0.04%
Penelope	-	-	0%	-	-	0%	-	-	0%
LINEs:	15,540	10,323,368	1.21%	21,102	7,010,014	1.06%	12,821	8,554,632	1.1%
CRE/SLACS	-	-	0%	-	-	0%	-	-	0%
L2/CR1/Rex	29	32,035	0%	452	115,253	0.02%	-	-	0%
R1/LOA/Jockey	253	27,544	0%	916	101,263	0.02%	-	-	0%
R2/R4/NeSL	114	31,454	0%	178	27,813	0%	599	307,317	0.04%
RTE/Bov-B	6,781	1,292,226	0.15%	6,183	1,004,976	0.15%	4,963	1,005,926	0.13%
L1/CIN4	7,922	8,874,721	1.04%	13,059	5,699,652	0.86%	7,259	7,241,389	0.93%
LTR elements:	256,535	256,951,419	30.16%	339,053	116,834,304	17.71%	246,773	231,236,242	29.79%
BEL/Pao	-	-	0%	1,240	225,945	0.03%	-	-	0%
Ty1/Copia	141,720	121,900,306	14.31%	189,531	66,582,347	10.09%	112,099	106,128,516	13.67%
Gypsy/DIRS1	102,476	118,892,264	13.96%	140,669	47,019,947	7.13%	114,587	107,691,852	13.87%
Retroviral	527	177,447	0.02%	288	48,620	0.01%	-	-	0%
DNA transposons	119,481	41,619,764	4.89%	104,864	22,729,296	3.45%	118,814	42,361,215	5.46%
hobo-Activator	67,134	22,901,196	2.69%	62,632	10,992,984	1.67%	64,423	22,739,074	2.93%
Tc1-IS630-Pogo	1,959	442,151	0.05%	3,299	519,231	0.08%	4,145	1,325,813	0.17%
En-Spm	-	-	0%	-	-	0%	-	-	0%
MuDR-IS905	-	-	0%	-	-	0%	-	-	0%
PiggyBac	-	-	0%	-	-	0%	-	-	0%
Tourist/Harbinger	19,749	5,232,736	0.61%	9,951	2,073,587	0.31%	8,457	2,261,836	0.29%
Other**	-	-	0%	-	-	0%	-	-	0%
Rolling-circles	7,614	6,403,344	0.75%	7,609	2,251,707	0.34%	6,932	7,395,035	0.95%
Unclassified:	763,966	173,474,545	20.36%	979,982	158,712,314	24.06%	694,716	157,723,725	20.32%
Total interspersed repeats:		482,916,602	56.69%		305,592,287	46.32%		440,184,678	56.71%
Small RNA:	6,911	1,206,922	0.14%	4,210	544,705	0.08%	4,655	848,344	0.11%
Satellites:	2,387	426,317	0.05%	1	343	0%	-	-	0%
Simple repeats:	141,867	6,073,335	0.71%	117,654	4,621,180	0.7%	138,316	5,699,855	0.73%
Low complexity:	25,393	1,212,646	0.14%	23,844	1,113,216	0.17%	23,018	1,078,243	0.14%

*most repeats fragmented by insertions or deletions have been counted as one element

**Mirage, P-element, Transib

F. nigra (Huff et al.)			*F. pennsylvanica*			*F. pennsylvanica* (Huff et al.)		
214,723			6,766			110		
625,073,830 bp (615,053,229 bp excl N/X-runs)			841,640,511 bp (841,635,311 bp excl N/X-runs)			756,791,283 bp (665,178,278 bp excl N/X-runs)		
34.54%			35.20%			34.40%		
306,699,285 bp (49.07%)			491,742,426 bp (58.43%)			344,208,795 bp (45.48%)		
# of elements*	length occupied (bp)	% of sequence	# of elements*	length occupied (bp)	% of sequence	# of elements*	length occupied (bp)	% of sequence
347,489	126,675,254	20.27%	292,123	261,510,864	31.07%	279,695	163,302,227	21.58%
1,683	231,103	0.04%	2,427	343,842	0.04%	5,436	648,994	0.09%
-	-	0%	-	-	0%	-	-	0%
18,999	6,031,961	0.96%	13,713	9,612,032	1.14%	15,367	7,398,417	0.98%
521	61,304	0.01%	-	-	0%	-	-	0%
115	39,869	0.01%	329	116,516	0.01%	488	76,648	0.01%
-	-	0%	-	-	0%	-	-	0%
-	-	0%	61	21,110	0%	-	-	0%
6,472	1,160,240	0.19%	4,947	902,746	0.11%	6,999	1,200,470	0.16%
11,710	4,747,814	0.76%	8,086	8,510,720	1.01%	7,003	5,785,517	0.76%
326,807	120,412,190	19.26%	275,983	251,554,990	29.89%	258,892	155,254,816	20.51%
43	26,727	0%	1,138	334,268	0.04%	2,142	333,257	0.04%
166,725	63,173,356	10.11%	136,320	118,623,599	14.09%	142,238	85,486,172	11.3%
151,873	53,757,778	8.6%	123,279	114,455,917	13.6%	104,707	64,798,318	8.56%
-	-	0%	49	23,025	0%	228	57,108	0.01%
112,004	24,162,089	3.87%	143,098	46,625,760	5.54%	108,249	29,043,246	3.84%
53,903	9,925,556	1.59%	67,546	23,718,166	2.82%	60,658	14,617,258	1.93%
2,188	457,866	0.07%	9,495	1,139,867	0.14%	2,374	573,558	0.08%
-	-	0%	-	-	0%	-	-	0%
-	-	0%	-	-	0%	-	-	0%
-	-	0%	-	-	0%	-	-	0%
17,062	3,659,059	0.59%	15,540	4,465,861	0.53%	14,509	3,152,855	0.42%
-	-	0%	320	85,516	0.01%	-	-	0%
7,830	2,271,092	0.36%	5,419	5,515,119	0.66%	3,597	2,299,028	0.3%
942,560	147,514,920	23.6%	781,229	168,274,085	19.99%	765,433	143,472,712	18.96%
	298,352,263	47.73%		476,410,709	56.61%		335,818,185	44.37%
5,859	747,714	0.12%	4,976	1,006,994	0.12%	8,579	1,226,042	0.16%
-	-	0%	6,447	1,206,761	0.14%	-	-	0%
120,232	4,541,894	0.73%	147,980	6,726,796	0.8%	115,169	4,434,286	0.59%
21,994	1,017,425	0.16%	24,652	1,195,549	0.14%	22,530	1,052,200	0.14%

Table S6: RagTag Stats for scaffolding long-read haploid Fraxinus assemblies using the *F. pennsylvanica* reference assembly from Huff et al.[1]

Assembly	placed_sequences	placed_bp	%_placed	unplaced_sequences	unplaced_bp	gap_bp	gap_sequences
F. americana	2747	812699353	95.40308324	1618	39159230	10631992	2718
F. nigra	1679	755230964	97.29115067	867	21027677	12972263	1652
F. pennsylvanica	4597	806317126	95.74463955	2182	35836680	13299209	4567

Table S7: Assembly and annotation Stats for *Fraxinus* assemblies generated from RagTag. *F. pennsylvanica* v1.4.1 is the chromosome-level assembly by Huff et al.[1] with the annotation generated in this study.

Assembly	*F. americana*	*F. nigra*	*F. pennsylvanica*	*F. pennsylvanica* v1.4
# contigs	1646	893	2210	110
Largest contig	56,786,594	54,652,814	56,064,724	56,547,140
Est. Total length	875 Mbp	829 Mbp	869 Mbp	869 Mbp
Total length	862,490,470	789,230,787	855,452,084	756,791,283
GC (%)	35.26%	34.76%	35.20%	34.40%
N50	36,855,385	34,535,975	35,808,976	33,221,578
L50	11	10	11	10
# N's per 100 kbp	1,233.50	1,644.13	1,555.25	12,120.86
Complete BUSCOs	97.3% (1570)	96.9% (1563)	96.8% (1562)	1576 (97.6%)
Complete single-copy BUSCOs	79.8% (1288)	80.7% (1302)	79.1% (1277)	1308 (81.0%)
Complete duplicated BUSCOs	17.5% (282)	16.2% (261)	17.7% (285)	268 (16.6%)
Fragmented BUSCOs	1.8% (29)	1.5% (24)	1.7% (27)	26 (1.6%)
Missing BUSCOs	0.9% (15)	1.6% (27)	1.5 (25)	12 (0.8%)
Total BUSCOs searched	1614	1614	1614	1614
**Annotation**	*F. americana*	*F. nigra*	*F. pennsylvanica*	*F. pennsylvanica* v1.4.1
gene model/mRNA count	41,166/46,547	37,509/42,801	40,546/45,811	43,842/48,640
Complete BUSCOs	97.5% (1573)	97.5% (1573)	98.2% (1585)	98.1% (1583)
Complete single-copy BUSCOs	79.1% (1276)	80.4% (1298)	79.6% (1284)	80.7% (1302)
Complete duplicated BUSCOs	18.4% (297)	17.0% (275)	18.6% (301)	17.4% (281)
Fragmented BUSCOs	0.8% (13)	0.8% (13)	0.8% (13)	1.1% (18)
Missing BUSCOs	1.7% (28)	1.7% (28)	1.0% (16)	0.8% (13)
Total BUSCOs searched	1614	1614	1614	1614

Table S8: Plant material and voucher collection details

Species collected	Latitude	Longitude	Location	Collection date	Sex	Voucher	Voucher date
F. pennsylvanica	42.48647	-79.35849	Point Gratiot Park; Dunkirk, New York, USA	30-May-21	Unknown	Yes	10-Jul-21
F. americana	42.34295	-79.42663	College Lodge Forest; Brocton, New York, USA	30-May-21	Unknown	Yes	10-Jul-21
F. nigra	42.34238	-79.42930	College Lodge Forest; Brocton, New York, USA	30-May-21	Unknown	Yes	10-Jul-21

References

1 Huff, M. *et al.* A high-quality reference genome for Fraxinus pennsylvanica for ash species restoration and research. *Molecular ecology resources* **22**, 1284-1302 (2022).
