# Supplementary material for: High quality genomes produced from single MinION flow cells clarify polyploid and demographic histories of critically endangered Fraxinus (ash) species
Source: Commun Biol. 2024 Jan 6;7:54. doi: 10.1038/s42003-023-05748-4 (PMC10771460; doi:10.1038/s42003-023-05748-4)
Supplement: Supplementary file 3 — Description of Additional Supplementary Files [file 42003_2023_5748_MOESM3_ESM.pdf]

Description of Additional Supplementary Files

File name: Supplementary Data 1
Description: Mutation rate correction for PSMCR
