# Supplementary material for: High quality genomes produced from single MinION flow cells clarify polyploid and demographic histories of critically endangered Fraxinus (ash) species
Source: Commun Biol. 2024 Jan 6;7:54. doi: 10.1038/s42003-023-05748-4 (PMC10771460; doi:10.1038/s42003-023-05748-4)
Supplement: Supplementary file 5 — Reporting Summary [file 42003_2023_5748_MOESM5_ESM.pdf]

nature portfolio

Corresponding author(s): Steven J. Fleck, Victor Al. Albert

Last updated by author(s): December 8, 2023

Reporting Summary

Nature Portfolio wishes to improve the reproducibility of the work that we publish. This form provides structure for consistency and transparency in reporting. For further information on Nature Portfolio policies, see our Editorial Policies and the Editorial Policy Checklist.

Statistics

For all statistical analyses, confirm that the following items are present in the figure legend, table legend, main text, or Methods section.

n/a	Confirmed	
☒	☐	The exact sample size (*n*) for each experimental group/condition, given as a discrete number and unit of measurement
☒	☐	A statement on whether measurements were taken from distinct samples or whether the same sample was measured repeatedly
☒	☐	The statistical test(s) used AND whether they are one- or two-sided<br>*Only common tests should be described solely by name; describe more complex techniques in the Methods section.*
☒	☐	A description of all covariates tested
☒	☐	A description of any assumptions or corrections, such as tests of normality and adjustment for multiple comparisons
☒	☐	A full description of the statistical parameters including central tendency (e.g. means) or other basic estimates (e.g. regression coefficient) AND variation (e.g. standard deviation) or associated estimates of uncertainty (e.g. confidence intervals)
☒	☐	For null hypothesis testing, the test statistic (e.g. *F*, *t*, *r*) with confidence intervals, effect sizes, degrees of freedom and *P* value noted<br>*Give P values as exact values whenever suitable.*
☒	☐	For Bayesian analysis, information on the choice of priors and Markov chain Monte Carlo settings
☒	☐	For hierarchical and complex designs, identification of the appropriate level for tests and full reporting of outcomes
☒	☐	Estimates of effect sizes (e.g. Cohen's *d*, Pearson's *r*), indicating how they were calculated

Our web collection on statistics for biologists contains articles on many of the points above.

Software and code

Policy information about availability of computer code

Data collection	No software was used for data collection
Data analysis	Guppy v5.0.11, Nanoplot v1.38.0, Nanostat v1.5.0, Flye v2.8.3, Flye v2.9, QUAST v5.0.2, BUSCO v5.4.4, GeMoMa v1.9, AGAT v1.0.0, Evidential Gene, RepeatModeler v2.0.1, RepeatMasker v4.0.1 , RagTag v2.1.0, MCScan (https://github.com/tanghaibao/jcvi/wiki/MCscan-(Python-version)), HapPy (https://github.com/AntoineHo/HapPy), SAMtools v1.14, Purge Haplotigs v1.1.1, Minimap2 v2.20, bcftools version 1.14, PSMCR (https://github.com/emmanuelparadis/psmcr), Orthofinder v2.5.4, Ksrates v1.1.3

For manuscripts utilizing custom algorithms or software that are central to the research but not yet described in published literature, software must be made available to editors and reviewers. We strongly encourage code deposition in a community repository (e.g. GitHub). See the Nature Portfolio guidelines for submitting code & software for further information.

Data

Policy information about availability of data

All manuscripts must include a data availability statement. This statement should provide the following information, where applicable:

- Accession codes, unique identifiers, or web links for publicly available datasets
- A description of any restrictions on data availability
- For clinical datasets or third party data, please ensure that the statement adheres to our policy

Raw reads for all species are available on the European Nucleotide Archive (ENA; https://www.ebi.ac.uk/). F. americana is under the study PRJEB47186 and raw

reads are sample accessions SAMEA9806828, SAMEA9806917, and SAMEA9806918. F. nigra is under the study PRJEB47212 and raw reads are sample accessions SAMEA9816504, SAMEA9816505, and SAMEA9816506. F. pennsylvanica is under the study PRJEB47234 and raw reads are sample accessions SAMEA9816501, SAMEA9816502, and SAMEA9816503. All assembly assembly versions generated in this study are available on the Comparative Genomics (CoGe) online platform (https://genomevolution.org/coge/). Initial Flye assemblies: F. americana v0.0 id66026, F. nigra v0.0 id66025, F. pennsylvanica v0.0 id66056; haploid-purged assemblies: F. americana v1 id66137, F. nigra v1 id66022, F. pennsylvanica v1 id66055; RagTag assemblies: F. americana v1Ragtag id66030, F. nigra v1Ragtag id66053, F. pennsylvanica v1Ragtag id66057; Reannotated Huff et al.17 assemblies: F. americana v0.2.1 id66014, F. nigra v0.2.1 id66015, F. pennsylvanica v1.4.1 id66054; Reannotated Forsythia suspensa v1.1 id66036; Reannotated Osmanthus fragrans v1.1 id66037. Genome assemblies, annotations, and source data for figures are available on Dryad (https://doi.org/10.5061/dryad.7sqv9s4xh). Supplementary Table 9 is available in Supplementary_Data_1.xlsx.

Research involving human participants, their data, or biological material

Policy information about studies with human participants or human data. See also policy information about sex, gender (identity/presentation), and sexual orientation and race, ethnicity and racism.

Reporting on sex and gender	NA
Reporting on race, ethnicity, or other socially relevant groupings	NA
Population characteristics	NA
Recruitment	NA
Ethics oversight	NA

Note that full information on the approval of the study protocol must also be provided in the manuscript.

Field-specific reporting

Please select the one below that is the best fit for your research. If you are not sure, read the appropriate sections before making your selection.

☐ Life sciences ☐ Behavioural & social sciences ☒ Ecological, evolutionary & environmental sciences

For a reference copy of the document with all sections, see nature.com/documents/nr-reporting-summary-flat.pdf

Ecological, evolutionary & environmental sciences study design

All studies must disclose on these points even when the disclosure is negative.

Study description	We carried out DNA sequencing, assembly, and annotation on three critically endangered Ash tree species (F. americana, F. nigra, and F. pennsylvanica). This study demonstrated what could be accomplished with sequencing an individual using a single Oxford Nanopore Technologies' (ONT) MinION flow cell. This included synteny and polyploid analysis, phylogenetics, and patterns in demographic history using ONT long-read data alone.
Research sample	Plant material and vouchers were collected from mature trees of unknown sex. Fraxinus pennsylvanica vouchers and material for DNA extraction were collected from Point Gratiot Park in Dunkirk, New York. F. americana and F. nigra vouchers and material for DNA extraction were collected from the College Lodge Forest in Brocton, New York.
Sampling strategy	Samples were chosen based on accessibility to living adult trees that could be identified in the field by Erik S. Danielson, Stewardship Coordinator for the Western New York Land Conservancy.
Data collection	The collections for material to extract DNA from took place on May 30, 2021, and vouchers from those same trees were collected on July 10, 2021. Fraxinus pennsylvanica vouchers and material for DNA extraction were collected from Point Gratiot Park in Dunkirk, New York. F. americana and F. nigra vouchers and material for DNA extraction were collected from the College Lodge Forest in Brocton, New York. From each sample, about 10g of leaf tissue was placed into separate vials, flash frozen using liquid nitrogen, and stored at -80 C for later use.
Timing and spatial scale	The material for DNA extraction was collected on a single day, May 30, 2021. This was due to DNA sequencing for genome assembly only requiring a relatively small amount of plant material. Leaves from each collection were flash frozen with liquid nitrogen and stored at -80 C later that same day.
Data exclusions	No exclusion of data
Reproducibility	Raw read data is publicly available for download. Using the software versions and running options as reported in the paper will reproduce our results.
Randomization	No clinical experimentation was done in the paper, therefore randomization was not needed.

Blinding	Blinding was not possible since the analyses did not include experimental vs. control groups.

Did the study involve field work? ☐ Yes ☒ No

Reporting for specific materials, systems and methods

We require information from authors about some types of materials, experimental systems and methods used in many studies. Here, indicate whether each material, system or method listed is relevant to your study. If you are not sure if a list item applies to your research, read the appropriate section before selecting a response.

Materials & experimental systems

n/a	Involved in the study
☒	☐ Antibodies
☒	☐ Eukaryotic cell lines
☒	☐ Palaeontology and archaeology
☒	☐ Animals and other organisms
☒	☐ Clinical data
☒	☐ Dual use research of concern
☐	☒ Plants

Methods

n/a	Involved in the study
☒	☐ ChIP-seq
☒	☐ Flow cytometry
☒	☐ MRI-based neuroimaging
